# Abstracts of Scottish Vision Group 2022 Meeting

**DOI:** 10.3390/vision6040057

**Published:** 2022-09-20

**Authors:** Jasna Martinovic, Nika Adamian, Mauro Manassi

**Affiliations:** 1 School of Philosophy, Psychology and Language Sciences, University of Edinburgh, Edinburgh EH8 9JZ, UK; 2 School of Psychology, University of Aberdeen, Aberdeen AB24 3FX, UK

**Keywords:** camouflage, visual cognition, locomotion, attention, eye movements, visual search, motion perception, multisensory perception

## Abstract

Since it was first launched in 2001, the Scottish Vision Group (SVG) has been a key meeting for vision scientists in Scotland, and has attracted vision scientists from the United Kingdom, Europe and beyond. This small conference is held annually at different places in Scotland. Its friendly atmosphere and stunning Scottish sceneries provide a great environment for relaxed scientific discussions. In particular, it is an excellent opportunity for scientists at an early stage of their career to give a talk about their work. The 2022 edition of SVG was held in St Leonard’s Hall at the University of Edinburgh. The meeting started with a panel discussion on camouflage led by Prof Nick Scott-Samuel (University of Bristol), Dr George Lovell (Abertay University) and Dr Rebecca Sharman (Abertay University). Research into camouflage has expanded remarkably over the last decade or so, with interdisciplinarity proving to be a key feature for progress. The discussion focussed on the different types of objectives and research techniques that are prominent in the field. The round table was sponsored by Meta Reality Labs. In the keynote lecture, sponsored by MDPI Vision, Prof Ute Leonards (University of Bristol) discussed the outcomes of her research programme investigating the crosstalk between visual cognition research and locomotion research. The outcomes of this Gibsonian approach do not just provide important insights into active vision but also outline the promising possibilities of sustainable urban design inspired by vision sciences. The rest of the conference was dedicated to talks on a variety of topics, including, but not limited to, attention, eye movements, visual search, motion perception, multisensory perception, colour and 3D vision. We present a selection of these abstracts. An associated Special Issue captures in fuller detail some of the research presented at SVG’s 2022 edition.

## 1. Round Table on Camouflage

George Lovell ^1^, Becky Sharman ^1^ and Nick Scott-Samuel ^2^

^1^ School of Applied Sciences, Abertay University
^2^ School of Psychological Science, University of Bristol

**Abstract:** Research into camouflage has exploded over the last decade or so, with interdisciplinarity proving to be a key feature for progress. In our round table, we will address three main questions: (i) why are we researching camouflage; (ii) how do we research camouflage; and (iii) what is camouflage, and how do we measure it? These are really just starting points for what we hope will be an informative, interactive and wide-ranging discussion. Gibson Revisited: toward understanding sensory affordances of real-world environments Ute Leonards (Ute.Leonards@bristol.ac.uk) School of Psychological Science, University of Bristol More than 50 years ago, JJ Gibson introduced the concept of affordances, which directly link actions to available information in our environments. Yet, even today, there is little crosstalk between visual cognition research and locomotion research, preventing us from understanding the affordances of sensory environments in real-world environments. In my talk, I will outline how following a Gibsonian approach could help us to find solutions to pressing societal issues such as understanding how the environments we create impact our health and wellbeing or how we could reduce fall risk in an ageing population. In a series of experiments, I will provide evidence of how the visual environment affects gait, even in hazard-free environments during walking on even ground—be it through the patterns on floor coverings or, more generally, the type of visual environment we are in. I will finish my talk with an outlook that goes beyond the research laboratory to modern urban design and will present a first attempt at a theoretical framework of Sustainable Urban Design informed by vision sciences.

## 2. The Multidimensional Spotlight of Attention

Søren K. Andersen ^1,2,^*

^1^ School of psychology, University of Aberdeen
^2^ Department of Psychology, University of Southern Denmark
* Correspondence: skandersen@health.sdu.dk

**Abstract:** The Spotlight and Zoomlens metaphors of attention have had, and still have, a profound influence on how researchers conceptualise and investigate visual selective attention. Since their development, subsequent research has shown that attentional selection is not just the product of a unitary mechanism for the selection of spatial locations but involves the joint operation of largely independent mechanisms that bias processing resources towards relevant information based on simple stimulus features such as colours, orientations, motion and spatial location. A substantial amount of research has considered these mechanisms individually. However, in more complex situations (e.g., real life) relevant stimuli may not be set apart from other stimuli by a single defining property, but by a specific combination of features. In such cases, effective attentional selection can only be achieved by concurrent selection of multiple features (e.g., feature conjunctions). To understand attention beyond artificial simplistic situations, it is therefore essential to know how attentional selection of different stimulus features is combined. In this talk, I will provide a synthesis of findings from a series of EEG experiments on feature-based and spatial attention and integrate them into an extended spotlight metaphor.

## 3. Deviations from Constancy in Contrast Matching over a Wide Range of Luminances

Maliha Ashraf ^1,^*, Sophie Wuerger ^1^, Jasna Martinović ^2^ and Rafał Mantiuk ^3^

^1^ Department of Psychology, University of Liverpool
^2^ School of Philosophy, Psychology and Language Sciences, University of Edinburgh
^3^ Department of Computer Science and Technology, University of Cambridge
* Correspondence: maliha.ashraf@liverpool.ac.uk

**Abstract:** The principle of contrast constancy in suprathreshold vision stipulates that the perception of contrast remains invariant across different parameters of stimuli, including spatial frequency, size, retinal eccentricity, etc. (Kulikowski, 1976 *Vision Research* 16 1419–1431). Contrast constancy is also generally believed to hold over changes in luminance levels (Peli, 1991 *JOSA A* 8 1352–1359), that is, stimuli with the same physical contrast but different mean luminances will have equal perceived contrast. However, in similar previous studies, only a limited range of luminances (1~2.6 log units) was used. Modern HDR displays are capable of displaying content ranging between 0.0001~1000 cd/m^2^. We investigate whether contrast constancy also holds when the matched stimuli vary across large dynamic ranges. We used two displays, each visible to only one eye with Gabor patches stimuli of three spatial frequencies with varying contrasts in the cardinal colour directions. The reference display showed a stimulus at a fixed luminance level of 200 cd/m^2^, whilst the test (HDR) display showed similar stimuli with luminance levels between 0.02–2000 cd/m^2^. Observers (n = 40) adjusted the contrast of the test stimuli until they perceived both the contrasts to be equal. We found that contrast constancy does not hold over such a large range of luminance levels (5 log units). Stimuli over 20 cd/m^2^ matched when their physical contrasts were the same for most conditions. However, for lower luminances, much higher physical contrast was required for equal contrast perception. This deviation from constancy was also larger for lower spatial frequencies and lower pedestal suprathreshold contrasts.

## 4. Modelling Target Selection Dynamics in Visual Foraging Tasks

Alasdair D F Clarke ^1,^*, Anna E Hughes ^1^ and Elia R Hunt ^2^

^1^ Department of Psychology, University of Essex
^2^ School of Psychology, University of Aberdeen
* Correspondence: a.clarke@essex.ac.uk

**Abstract:** In visual foraging tasks, participants must find as many targets as they can. The targets are typically hidden among distracters and there are multiple different classes of targets. Previous work (Kristjánsson, et al., 2014, *PloS ONE* 9.6) has demonstrated that when the targets are defined in terms of a conjunction of features, participants tend to select targets in a small number of long runs, typically selecting most of the targets of one class before switching to another. This contrasts with single feature foraging, in which participants are much more likely to switch from one target class to another. In our recent work, we presented a generative model for target selection behaviour in this task. In this talk, I will give examples of how this modelling framework can be used to further our understanding of foraging behaviour. For example, when the task is made more realistic by using a 3D virtual reality environment (Prpic, et al., 2019, *PloS ONE* 14.7), participants place more weight on selecting targets that lie ahead of them. We also demonstrate that the distinction between feature and conjunction conditions can generalise to easy and hard conditions. Finally, we examine how stopping rules could be implemented in the model.

## 5. Using Perceptual Aftereffects to Reveal the Underlying Mechanisms of Orientation Perception

Nikos Gekas *

Department of Psychology, Edinburgh Napier University
* Correspondence: n.gekas@napier.ac.uk

**Abstract:** Visual perception is a perpetually adapting process where the current percept is affected by what has been previously observed. It has been shown that stimuli seen at different times in the past can have differing effects on the perceived features (e.g., orientation) of the current stimulus (Gekas et al., 2019, Journal of Vision 19 24–24). I will present an orientation discrimination task in which systematic manipulation of the contrast of past stimuli reveals a strong interaction between contrast and perceptual aftereffects, and suggests a complex relation between stimulus orientation, contrast, duration and time of presentation. I will discuss how these experimental findings and computational modelling can help us understand the underlying mechanisms that are responsible for the encoding and decoding of visual information.

## 6. Developing a Collaborative Framework for Naturalistic Visual Search

Anna E. Hughes ^1,^*, Kenneth C. Scott-Brown ^2^, Charli Sherman ^1^, Angela Kokic Brnicevic ^1^, Alice Mariuzzo ^1^ and Alasdair D. F. Clark ^1^

^1^ Department of Psychology, University of Essex
^2^ Division of Psychology and Forensic Sciences, Abertay University
* Correspondence: anna.hughes@essex.ac.uk

**Abstract:** While much research has investigated the mechanisms of visual search behaviour in laboratory-based computer tasks, there has been relatively little work on whether these results generalise to more naturalistic search tasks and thus how well existing theories explain real-world search behaviour. In addition, work relating to this question has often been carried out by researchers working in very different disciplines, including not just vision science but also fields such as consumer behaviour, sports science and medical science, making it more difficult to get an overview of the progress made and open questions remaining. We present findings from a systematic review of real-world visual search, showing that we can group the current literature into theoretical and applied approaches, and that there are certain well-studied topics (e.g., X-ray screening) but that there are relatively few links made across different search tasks and/or search contexts. We also present preliminary work detailing our development of a “naturalistic search task battery”, which aims to provide a suite of open source, reproducible and standardised real-world search tasks, thus enabling the generation of comparable data across multiple studies and aiding theory and modelling in this area.

## 7. The Relationship between Strategies and Performance in Visual Search

Letizia Caruso ^1^, Anna Nowakowska ^1^, Alasdair D.F. Clarke ^2^ and Amelia R. Hunt ^1,^*

^1^ School of Psychology, University of Aberdeen
^2^ School of Psychology, University of Essex
* Correspondence: a.hunt@abdn.ac.uk

**Abstract:** Efficient eye movements are those directed to regions where central vision is most needed, relative to locations that could be evaluated using peripheral vision. We previously observed large individual differences in efficiency and found that making inefficient eye movements early on target-absent trials is strongly correlated with being slower to find targets when they are present. In the current study, we re-analysed previous datasets to look at the relationship between inefficient fixations and other kinds of eye movement behaviours, including revisiting previously-inspected locations and looked-but-failed-to-see errors (where the participant looks directly at the target and then reports it as absent). We also categorised scanpaths according to whether they contained a systematic strategy, such as a left-right and top-bottom “reading” of the search array, versus being apparently haphazard. For all of these measures of eye movement strategies, what emerges is a complex set of trade-offs between search speed, working memory load and error tolerance. These trade-offs vary a great deal from one person to another and can have large effects on search speed.

## 8. High-Level Effects in Crowding Cannot Be Explained by High-Dimensional Pooling

Mauro Manassi *

School of Psychology, University of Aberdeen, UK
* Correspondence: mauro.manassi@abdn.ac.uk

**Abstract:** In crowding, target perception deteriorates when flanking elements are added. Crowding is traditionally characterised by low-level target-flanker interactions which are deleterious, spatially confined within Bouma’s window and feature-specific. Crucially, information lost in the early stages is irretrievably lost. Recently, a vast literature on high-level effects in crowding (grouping effects and face-holistic crowding in particular) led to a different understanding of crowding, as a global, complex and multilevel phenomenon that cannot be captured or explained by simple pooling models. It was recently argued that these high-level effects may still be captured by more sophisticated pooling models, such as the Texture Tiling model. Here, I extensively tested the predictions of the Texture Tiling model on the results of six different studies that highlighted high-level effects in crowding. The results show that the Texture Tiling model cannot explain any of these high-level effects and that the behaviour of the model is equivalent to a simple pooling model. Taken together, these results reinforce the idea that complex target-flanker interactions determine crowding and that crowding occurs at multiple levels of the visual hierarchy.

## 9. Ensemble Perception of First Impressions of Trustworthiness

Fiammetta Marini *, Clare Sutherland and Mauro Manassi

School of Psychology, University of Aberdeen
* Correspondence: f.marini.21@abdn.ac.uk

**Abstract:** Trustworthiness is a fundamental social judgment with deep consequences in society. Most research has focused on individual facial characteristics that make a face more or less trustworthy. However, in everyday life faces are not always perceived in isolation but are often encountered in crowds. It has been proposed that our visual system deals with a large amount of facial information in a group by extracting summary statistics of the crowd—a phenomenon called ensemble perception. Prior research showed that ensemble perception occurs for various facial features, such as emotional expression, facial identity and attractiveness. Here, we investigated whether observers are able to extract an ensemble percept of trustworthiness from multiple faces. In this study, participants were presented with crowds of faces varying in the level of trustworthiness and were asked to adjust a subsequent face by scrolling through a morphed continuum to match the average level of trustworthiness of the previously seen group. To rule out subsampling, we tested ensemble recognition when only 1, 2 or 4 faces were displayed. These control conditions allowed us to simulate what participants’ estimates would have been if they had randomly subsampled a subset of faces from the crowd. We measured participants’ absolute errors across the set size conditions and found that participants increasingly integrated trustworthiness information from our set of faces. These results suggest that observers successfully extracted the average trustworthiness level from the whole sets of faces. Taken together, this experiment demonstrated that ensemble perception occurs at the level of first impressions of trustworthiness.

## 10. Decoding of Neural Activity from MEEG Signals Is Highly Sensitive to Latency Shifts

Jasna Martinovic *

School of Philosophy, Department of Psychology, Psychology and Language Sciences, University of Edinburgh
* Correspondence: jmartino@ed.ac.uk

**Abstract:** Decoding from magneto and electroencephalographic (MEEG, for short) signals is quickly gaining in prominence in visual neuroscience. This method of analysis can provide insights into the nature of the neurometric space in which various perceptual features are represented. Decoding from MEEG signals is commonly done by dividing and averaging single trials into several folds – thus creating a set of several relatively noisy event-related potentials (ERPs). Information decoding is then performed sample by sample from a multidimensional space in which all channels act as features. Decoding from MEEG signals works remarkably well considering the fact that their spatial resolution is much inferior to functional magnetic resonance imaging (fMRI). This may appear surprising, considering that decoding is done on spatially distributed features (i.e., electrode channels). However, superior decoding in MEEG does not necessarily have to reflect the spatial distribution of signals. In this study, I simulate two ERP signals that differ only in the latency of the peak of the P300a component. Peaks are always projected from the standard P300a source, with a fixed location and orientation. This effectively results in the same signal in the topographical activity map, which is shifted in time. The two conditions are decodable above chance despite the fact that they do not involve any systematic spatial alteration in the signal. This is evidence that temporal activation patterns cannot be neglected when interpreting outcomes of decoding analyses in visual neuroscience, in particular as multiple stimulus modulations are known to elicit temporal shifts of signal (e.g., contrast-dependent latency shifts).

## 11. Irrelevant Surface Level Properties Modulate Visual Search Efficiency

Anna Nowakowska ^1,^*, Alasdair D.F. Clarke ^2^, Josephine Reuther ^3^ and A.R. Hunt ^1^

^1^ Department of Psychology, University of Aberdeen
^2^ Department of Psychology, University of Essex
^3^ Department of Experimental Psychology, University of Goettingen
* Correspondence: a.nowakowska@abdn.ac.uk

**Abstract:** Because of the limitations of our foveated visual system (high-resolution information can only be sampled sequentially), the efficiency of our eye movements in targeting locations where high resolution is needed influences the speed of visual search. Some models suggest humans engage in optimal search; others imply human search is best described by a stochastic process. Across six experiments, we measured efficiency of healthy observers (E1 = 30, E2 = 15, E3 = 15, E4 = 15, E5 = 30, E6 = 15) as they searched through line segment stimuli, computer desktop icons, mosaic patterns, simple polygons and pens. The search array was always split vertically into easy and hard sides. When the target was present on the easy side it was detectable in peripheral vision and hence observers need not move their eyes to detect it. When the target was present on the hard side, it was not detectable in peripheral vision and so the most efficient search strategy was directing fixations to this side. This optimal strategy, in principle, is the same and no more or less difficult to implement across the stimulus sets. Nonetheless, strikingly different patterns of results emerged across different types of stimuli, which hinged largely on how the target of the search was defined. Searching for an object oriented in a particular direction produced highly variable efficiency that, on average, did not differ from what would be expected from a stochastic strategy. In comparison, searching for a specified object produced far more uniform and efficient search behaviour. The results demonstrate that small changes to seemingly irrelevant details of the task can lead to large changes in measured strategy and performance. Moreover, searching for visual features is a useful and common laboratory task, but it may not be representative of the search for whole objects.

## 12. Effects of Spatial Arrangement of Flankers in the Eriksen Flanker task

Danai Papadaki ^1,^*, Rama Chakravarthi ^1^ and ren K. Andersen ^1,2^

^1^ School of Psychology, University of Aberdeen
^2^ Department of Psychology, University of Southern Denmark
* Correspondence: d.papadaki.19@abdn.ac.uk

**Abstract:** Interference from task-irrelevant visual stimuli (“flankers”) has predominantly been studied separately in the Eriksen flanker task and visual crowding, despite obvious similarities between the two approaches. In order to explore possible similarities or demarcations between them, we examined whether well-established effects of the spatial arrangement of flankers on crowding also produce a comparable impact on congruency effects in the flanker task, even under conditions of no crowding. Specifically, we tested (1) a radial-tangential anisotropy, (2) an inner-outer flanker asymmetry and (3) an effect of number of flankers. As in crowding, we found that the congruency effect (improved performance in congruent compared to incongruent trials) in the Eriksen flanker task was stronger for radial compared to tangential flankers. A greater congruency effect was also observed when two flankers were presented compared to one. In addition, we found an inner-outer asymmetry where the congruency effect was stronger in the presence of an inner flanker compared to an outer flanker, in apparent contradiction with a key signature of crowding. Our finding could be considered in relation to Strasburger’s reports that the inner flanker is more often confused with the target in crowding tasks. We conclude that the differential influence of flanker location on the congruency effect provides evidence towards an underlying mechanism of flanker interference that is present both at the visual processing and the response selection stages. These results suggest that the two approaches might be more closely related than originally thought.

## 13. A Rapid and Efficient Source Localisation Method Using Functionally Defined EEG Templates

Marlene Poncet * and Justin Ales

School of Psychology & Neuroscience, University of St Andrews
* Correspondence: mfp7@st-andrews.ac.uk

**Abstract:** Electroencephalography (EEG) is a common and relatively cheap method to record neural activity in humans. However, it lacks spatial resolution making it difficult to determine which areas of the brain are responsible for the observed EEG response. Here, we present a new easy-to-use method using functionally defined EEG templates to determine which brain areas contribute to the EEG response. We collated anatomical and functional MRIs of 50 participants comprising both retinotopic and functional localisers. From this data, we simulated how sources in visual areas (V1, V2, V3, V4, V3A, LOC, hMT+) appear on the scalp and averaged this signal across participants to produce functionally defined EEG templates. These templates can then be used via linear regression to estimate how much each visual area contributes to the observed EEG activity. We tested this new method using real and simulated data while manipulating the signal-to-noise ratio, number of participants, electrode montage and number of brain areas active at the same time. The proposed procedure accurately recovers signals in the active brain areas and is as well as bespoke individual source localisation methods do. It can be used on any EEG dataset, past or present. This new template-based method has multiple strengths: it is easy to understand, simple to use, has low computational demand and does not involve additional resources (fMRI scans). We thus expect it to be of wide interest to EEG users.

## 14. In Vision, It Is Groups, Rather Than Maps, That Determine How We Perceive the World

Philip Quinlan ^1,^*, Keith Allen ^2^ and Dale J. Cohen ^3^

^1^ Department of Psychology, University of York
^2^ Department of Philosophy, University of York
^3^ Department of Psychology, University of North Carolina, Wilmington
* Correspondence: philip.quinlan@york.ac.uk

**Abstract:** It is generally acknowledged that visual dimensions, such as colour and form, are processed separately in early vision. In dispute, however, is whether these dimensions are functionally independent. Boolean Map (BM) Theory assumes a strong form of independence between separate types of features, such that processing is restricted to consideration of only one feature at a time. As a consequence, type distinct features must be processed sequentially. In contrast, theories that emphasise the early grouping of features, such as discussed in the Gestalt tradition, assume feature processing is interactive. The interaction between features in early vision is seen to underpin figure/ground segregation as a necessary precursor to object perception. To test between such ideas, we report performance in a speeded counting task in which displays contained squares and circles that appeared in either of two colours. The task was to judge which shape was more prevalent. Importantly, the colour and shape distinctions could be perfectly correlated (i.e., compatible) or not (i.e., incompatible). BM theory predicts no influence of the relative coincidence of colour and shape on the identification of the more prevalent shape. In contrast, grouping theory predicts that performance will be better when the colour/shape distinction is compatible than when it is incompatible. Our data strongly support the grouping theory predictions. We conclude that a primary constraint in vision is the number and kind of groupings that are recovered rather than the number of feature maps consulted.

## 15. Role of Edge in Appearance of Saturation as a Function of Stimulus Size

Ana Rozman ^1,^* and Jasna Martinovic ^2^

^1^ School of Psychology, University of Aberdeen
^2^ Department of Psychology, School of Philosophy, Psychology and Language Sciences, University of Edinburgh
* Correspondence: a.rozman@abdn.ac.uk

**Abstract:** Parafoveally presented chromatic stimuli have previously been found to appear desaturated when their size is reduced (e.g., Knau & Werner, 2002, JOSA A, 19, 208–214). We replicated this effect using isoluminant stimuli defined along the cardinal axes of the cone-opponent colour space (bluish, yellowish, reddish and greenish). Whilst the effect was stronger for older compared to younger observers, both groups experienced the strongest desaturation for bluish, S- cone isolating stimuli. To evaluate the mechanisms driving this asymmetry, we examined the importance of stimulus edge to saturation perception. A group of younger observers adjusted the chromatic contrast of the four cardinal colours differing in size to match the saturation of a standard 2° stimulus. We varied edge definition by presenting stimuli on positive and negative polarity luminance pedestals, set at magnitudes of 3 or 6 individually measured just-noticeable differences in brightness (JNDs). The standard stimulus was presented either with a sharp or blurred edge. Desaturation with reduced stimulus size was lower only in the presence of negative luminance polarity pedestals, demonstrating an asymmetry in how the two polarities combine with colour contrast. Meanwhile, desaturation remained the strongest for bluish stimuli. As expected, blurring of the standard stimulus edge reduced the overall perceived contrast for the 2° stimulus but it did not change the effect of stimulus size. It appears that rather than edge sharpness, it is parafoveal contrast sensitivity that determines perceived contrast. On relatively small luminance pedestals, such as those used here, the contrast sensitivity remains driven by the chromatic content of the stimulus.

## 16. In a Case of Longstanding Low Vision Regions of Visual Cortex That Respond to Tactile Stimulation of the Finger with Braille Characters Are not Causally Involved in the Discrimination of those Same Braille Characters

Edward H. Silson ^1,2,^*, Andre D. Gouws ^3^, Gordon E. Legge ^4^ and Antony B. Morland ^2,3,5^

^1^ Department of Psychology, School of Philosophy, Psychology and Language Sciences, The University of Edinburgh
^2^ Department of Psychology, University of York
^3^ York Neuroimaging Centre, The Biocentre, York Science Park
^4^ Department of Psychology, University of Minnesota
^5^ York Biomedical Research Institute, University of York
* Correspondence: ed.silson@ed.ac.uk

**Abstract:** Braille reading, and other tactile discrimination tasks, recruit the visual cortex of both blind and normally sighted individuals undergoing short-term visual deprivation. Prior functional magnetic resonance imaging (fMRI) work on patient “S”, a visually impaired adult with the rare ability to read both highly magnified print visually and Braille by touch, found that foveal representations of S’s visual cortex were recruited during tactile perception, whereas peripheral regions were recruited during visual perception (Chueng et al., 2009). Here, we test the causal nature of tactile responses in the visual cortex of S by combining tactile and visual psychophysics with repetitive transcranial magnetic stimulation (rTMS). First, we replicate the previous fMRI findings in S. Second, we demonstrate that transient disruption of S’s foveal visual cortex has no measurable impact on S’s tactile processing performance compared to that of healthy controls—a pattern not predicted by the fMRI results. Third, stimulation of the foveal visual cortex maximally disrupted visual processing performance in both S and controls, suggesting the possibility of preserved visual processing within S’s foveal representation. Finally, stimulation of the somatosensory cortex induced the expected disruption to tactile processing performance in both S and controls. These data suggest that tactile responses in S’s foveal representation reflect the unmasking of latent connections between visual and somatosensory cortices and not behaviourally relevant cross-modal plasticity. Unlike studies in congenitally blind individuals, it is possible that the absence of complete visual loss in S has limited the degree of causally impactful cross-modal reorganisation.

## 17. Virtual Reality: A Tool for Investigating Camouflage

Ioan E. Smart *, Rebecca J. Sharman, Kenneth C. Scott-Brown and P. George Lovell

School of Applied Sciences, Abertay University
* Correspondence: i.smart1900@abertay.ac.uk

**Abstract:** Disruptive camouflage utilises high-contrast patches, typically positioned at the margins of an object to impede the detection and/or recognition of a perceiver. To date, the predominant methods for examining camouflage strategies are computer-based (i.e., detection experiments), field-based (e.g., survival analyses) and camouflage choice experiments using dynamically coloured organisms (e.g., cephalopods). Recent advances in virtual reality (VR) technology present the opportunity to create novel environments for testing camouflage theory. VR can combine the control of lab-based research with the ecological validity of field-based studies. Here, we develop an experimental paradigm that enables camouflage testing within a virtual reality environment. The environment comprised a spherical target that can be wrapped with different camouflage patterns and a domed background, upon which a natural image can be projected. Participants were positioned within the centre of the dome and were tasked with finding and shooting at targets randomly positioned across a bounded range within the environment. We manipulated the luminance contrast (0–2 steps of 2.5 L *) of disruptive and edge-enhancement (EE) components of the camouflage patterning to examine their impact on participant response time. Having high, but not extreme, contrast resulted in increased camouflage effectiveness. The EE component had no effect independently but interacted with the DC component. Specifically, when using EE alongside DC, a lower contrast EE component is more effective than a higher contrast EE component. Our results demonstrate that VR is a viable research tool for testing camouflage theory.

